# 233. Safety of Ceftazidime-Avibactam (CZA) in Combination with Aztreonam (ATM) in a Phase I, Open-Label Study in Healthy Adult Subjects (COMBINE)

**DOI:** 10.1093/ofid/ofac492.311

**Published:** 2022-12-15

**Authors:** Thomas Lodise, J Nicholas O’Donnell, Shruti Raja, Stephen Balevic, Jeffrey Guptill, Nyssa Schwager, Smitha Zaharoff, Vance G Fowler, Varduhi Ghazaryan, Tatiana Beresnev, Alison wall, Katherine Wiegand, Henry Chambers

**Affiliations:** Albany College of Pharmacy and Health Sciences, Albany, New York; Albany College of Pharmacy and Health Sciences, Albany, New York; Duke Early Phase Clinical Research Unit, Duke Clinical Research Institute (DCRI), Duke University School of Medicine, Durham, North Carolina; Duke Clinical Pharmacometrics Center, Duke Clinical Research Institute (DCRI), Duke University School of Medicine, Durham, North Carolina; Duke University School of Medicine, Durham, North Carolina; Duke Clinical Research Institute (DCRI), durham, North Carolina; Duke Clinical Research Institute (DCRI), durham, North Carolina; Duke University Medical Center, Durham, North Carolina; Division of Microbiology and Infectious Diseases (DMID), National Institute of Allergy and Infectious Diseases (NIAID), National Institutes of Health (NIH), Bethesda, Maryland; Division of Microbiology and Infectious Diseases (DMID), National Institute of Allergy and Infectious Diseases (NIAID), National Institutes of Health (NIH), Bethesda, Maryland; The Emmes Company, Rockville, Maryland; The Emmes Company, Rockville, Maryland; University of California, San Francisco, and San Francisco General Hospital, San Francisco, California

## Abstract

**Background:**

The combination of CZA-ATM is frequently used to treat patients with metallo-β-lactamase (MBL)-producing Enterobacterales (EB) infections, but its safety has not been established in controlled trials. This phase 1 study evaluated the safety of the optimal CZA-ATM regimens identified in the hollow fiber infection model of MBL-producing EB (PMID: 32464664).

**Methods:**

The phase I, open-label, single center study enrolled healthy adults aged 18-45 years (NCT03978091). Subjects were sequentially assigned to 1 of 6 Cohorts and administered investigational product(s) (IP) for 7 days (**Table 1**). Study safety was monitored by assessments of adverse events (AEs), vital signs, and clinical laboratory safety tests.

**Figure:**
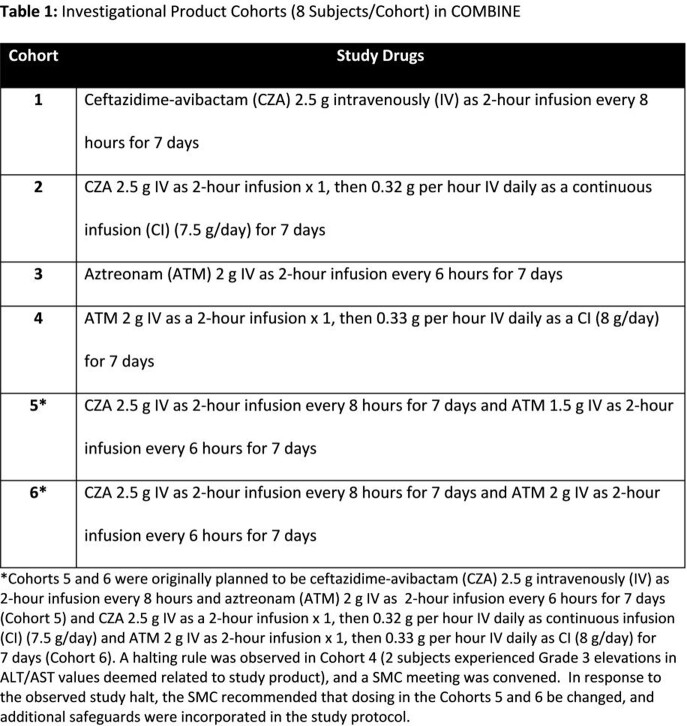

**Results:**

Of 48 subjects enrolled, 50% were female and 60% were Black. The mean (SD) age was 33.5 (6.2) years and mean (SD) weight was 75.7 (12.1) kg. The number of subjects who had ≥ 1 AE and experienced ≥ 1 IP related AE was 46 (96%) and 41 (85%), respectively. Frequency of IP related AEs by MedDRA system organ class, severity, and Cohort are shown in **Figure 1.** The occurrence of IP related investigation AEs were more frequent in the combination vs single IP Cohorts (**Table 2**). The most common IP related investigation AEs were ALT/AST elevations (35%) with 94% occurring in subjects who received ATM alone or in combination. The incidence of ALT/AST elevation AEs in the combination Cohorts were comparable to the ATM alone Cohorts. In the ATM single IP Cohorts, 3 subjects experienced severe ALT/AST elevation AEs, which halted the study. All subjects who experienced ALT/AST elevations were asymptomatic, had no other clinical findings suggestive of liver injury, and all resolved without intervention. Most other IP related AEs were of mild severity and similar across Cohorts except prolonged prothrombin time (PT) AEs, which was more frequent in combination Cohorts.

**Figure:**
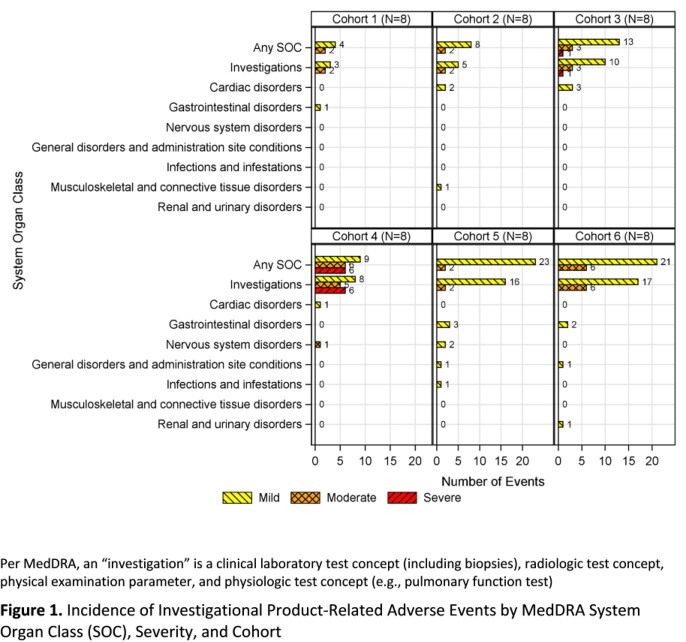

**Figure:**
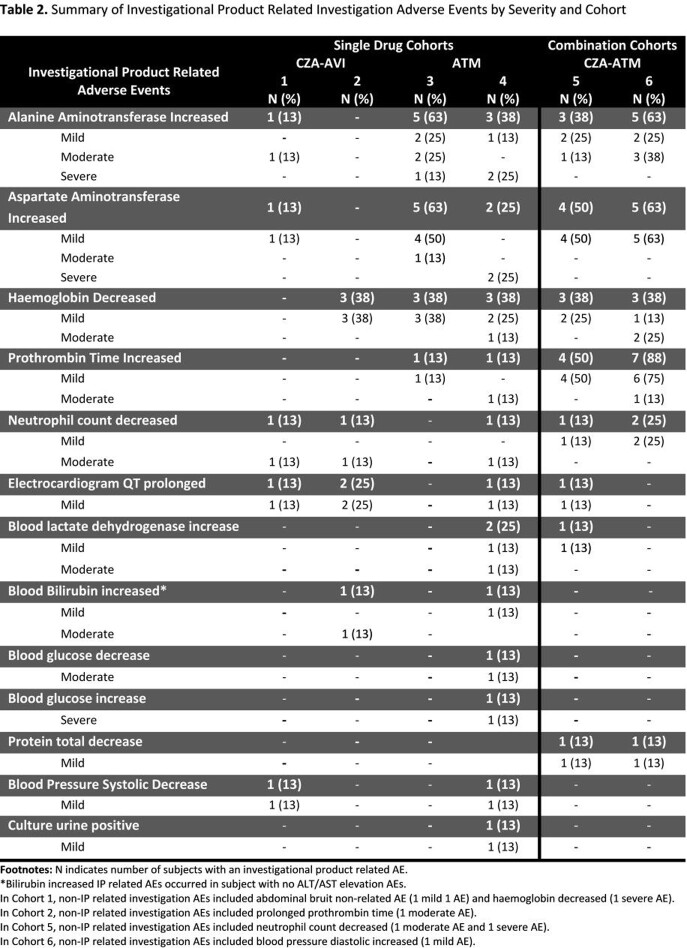

**Conclusion:**

Clinicians should only consider using CZA-ATM when the benefits outweigh the risks. If CZA-ATM is prescribed, clinicians are advised to monitor for hepatic injury. Close monitoring of coagulation parameters may also be prudent with CZA-ATM. Future comparator-controlled randomized clinical trials are required to better define the safety and efficacy of the CZA-ATM regimens.

**Disclosures:**

**Thomas Lodise, Jr., Pharm.D., PhD**, BioFire Diagnostics: Grant/Research Support|cidara: Advisor/Consultant|cidara: Honoraria|Entasis: Grant/Research Support|Merck: Advisor/Consultant|Merck: Grant/Research Support|Paratek: Advisor/Consultant|Shionogi: Advisor/Consultant|Spero: Advisor/Consultant|Venatrox: Advisor/Consultant **J Nicholas O'Donnell, Pharm.D.**, Merck & Co, Inc: Grant/Research Support|Paratek Pharmaceuticals: Grant/Research Support **Stephen Balevic, MD**, Purdue Pharma: Grant/Research Support|UCB: Advisor/Consultant **Jeffrey Guptill, MD**, argenx: Stocks/Bonds **Vance G. Fowler, Jr, MD, MHS**, Affinergy: Grant/Research Support|Affinergy: Honoraria|Affinium: Honoraria|Amphliphi Biosciences: Honoraria|ArcBio: Stocks/Bonds|Basilea: Grant/Research Support|Basilea: Honoraria|Bayer: Honoraria|C3J: Honoraria|Cerexa/Forest/Actavis/Allergan: Grant/Research Support|Contrafect: Grant/Research Support|Contrafect: Honoraria|Cubist/Merck: Grant/Research Support|Debiopharm: Grant/Research Support|Deep Blue: Grant/Research Support|Destiny: Honoraria|Genentech: Grant/Research Support|Genentech: Honoraria|Integrated Biotherapeutics: Honoraria|Janssen: Grant/Research Support|Janssen: Honoraria|Karius: Grant/Research Support|Medicines Co.: Honoraria|MedImmune: Grant/Research Support|MedImmune: Honoraria|NIH: Grant/Research Support|Novartis: Grant/Research Support|Novartis: Honoraria|Pfizer: Grant/Research Support|Regeneron: Grant/Research Support|Regeneron: Honoraria|Sepsis diagnostics: Sepsis diagnostics patent pending|UpToDate: Royalties|Valanbio: Stocks/Bonds **henry chambers, MD**, Merck: DSMB member|Merck: Stocks/Bonds|Moderna: Stocks/Bonds.

